# Periodically rotated overlapping parallel lines with enhanced reconstruction acquisition to improve motion-induced artifacts in bladder cancer imaging: Initial findings

**DOI:** 10.1097/MD.0000000000017075

**Published:** 2019-10-18

**Authors:** Huyen Thanh Nguyen, Zarine Ketul Shah, Amir Mortazavi, Kamal S. Pohar, Lai Wei, Debra Lyn Zynger, Michael Vinzenz Knopp

**Affiliations:** a Wright Center of Innovation in Biomedical Imaging, Department of Radiology; b Department of Internal Medicine; c Department of Urology; d Center for Biostatistics; e Department of Pathology, The Ohio State University, Columbus, OH.

**Keywords:** bladder cancer, motion-induced artifacts, PROPELLER acquisition, T2W-MRI

## Abstract

Motion-induced artifacts have been a major drawback in bladder cancer imaging. This study is to evaluate the clinical utility of periodically rotated overlapping parallel lines with enhanced reconstruction (PROPELLER) acquisition in improving motion-induced artifacts in T2-weighted (T2W) magnetic resonance imaging (MRI) of bladder cancer at 3T.

Sixteen patient MRI exams were included. Using a Likert scale, 2 radiologists independently scored T2W data without and with PROPELLER in terms of artifact severity and tumor visualization. Statistical analysis was done to assess the image quality improvement by PROPELLER and inter-observer variability.

Without PROPELLER, the median scores of artifact severity and tumor visualization were 1.5 and 1.5 for reviewer 1, and 2.0 and 2.0 for reviewer 2. With PROPELLER, the scores increased to 3 and 3.5 for reviewer 1, and 3.5 and 3.5 for reviewer 2. Despite the inter-observer variability (*κ* scores < 0.2), both reviewers found significant improvement in artifacts and visualization (all *P* < .001).

PROPELLER acquisition significantly improved the image quality of T2W-MRI. These initial findings indicate that this technique should be utilized in clinical MRI of the bladder.

## 1 Introduction

Transitional cell carcinoma accounts for about 90% of bladder cancer cases. The invasion depth of urothelial cancers into the bladder wall is a critical diagnostic factor in risk and treatment stratification.^[1]^ While nonmuscle invasive bladder cancer can be treated with a preservative approach including intravesical chemotherapy and immunotherapy, the definitive treatment for muscle-invasive bladder cancer (MIBC) is radical cystectomy or radiotherapy following recommended neoadjuvant chemotherapy (NAC). Therefore, it is clinically important in bladder imaging to provide the delineation of bladder tumors against the bladder wall. Magnetic resonance imaging (MRI) with high soft-tissue contrast has high potential to meet this clinical need in bladder cancer management. However, due to a long data acquisition, motion artifacts have been a hindrance to high image quality MR images. In pelvic imaging including bladder imaging, respiration and peristalsis are unavoidable motion which degrade image quality and may cause diagnostic inaccuracy.^[2]^

Recently, a non-Cartesian k-space sampling technique, called periodically rotated overlapping parallel lines with enhanced reconstruction (PROPELLER), has become available to correct motion artifacts in MRI. This method samples k-space in rotating strips of multiple phase-encoding lines. This technique over-samples the center of k-space and discards inconsistent data, which are associated with the motion. PROPELLER acquisition has been shown to substantially improve or eliminate motion artifacts for a number of clinical applications including brain, shoulder, upper abdomen, and cardiac imaging.^[3–17]^ To date, there has been no reported data about the value of PROPELLER in bladder cancer imaging.

This study aimed to evaluate the clinical utility of the PROPELLER technique in MRI to reduce motion artifacts in morphologic T2-weighted (T2W) imaging of bladder cancer at 3T.

## 2 Methods

### 2.1 Subjects

This study is part of a prospective study to improve the diagnosis and chemotherapeutic monitoring of bladder cancer. The Institutional Review Board approved the study protocol. Patient enrollment criteria include:

(1) pathological reports confirmed MIBC (pT2);
(2) NAC has been scheduled;
(3) radical cystectomy is planned after NAC;
(4) there are no MRI contraindications.

All patients provided written informed consent before their enrollment.

Since this prospective study was initiated in 2009, we had observed substantial motion artifacts present in about 30% of our patient MRI exams. Thus, when PROPELLER became available in our scanner, from June 2015 to July 2018, we started applying the technique to improve motion-induced artifacts seen on T2W images. The T2W-MRI with PROPELLER was performed immediately after the initial T2W scan. Sixteen bladder MRI exams have been performed with PROPELLER and included in this assessment.

### 2.2 MRI protocol

All scans were performed on a 3T Ingenia CX (Philips Healthcare, Cleveland, Ohio). Both T2W MRI scans with and without PROPELLER were acquired with turbo spin-echo sequence. The imaging parameters of T2W without MultiVane, the vendor acronym of PROPELLER, were repetition time/echo time (TR/TE), 5480/91 ms; matrix, 100/140; in-plane field of view (FOV) (anterior-posterior [AP]/right-left [RL]), 300/340 mm; slice thickness. 3.0 mm; slice gap, 0.3 mm; in-plane resolution, 0.5 × 0.5 (mm); number of slices, 45; number of signal average, 1; sensitivity encoding (SENSE) factor, 2; scan time, 285 seconds. The parameters with MultiVane were TR/TE, 6278/125 ms; in-plane FOV (AP/RL), 300/300 mm; slice thickness, 3.0 mm; slice gap, 0.3 mm; in-plane resolution, 0.5 × 0.5 (mm); number of slices, 45; number of signal average, 1; SENSE factor, 3; scan time, 327 seconds; MultiVane factor, 360%.

### 2.3 Data analysis

Two radiologists (with 15 and 30 years of experience) independently reviewed T2W data first without, then with MultiVane to assess the image quality for diagnosis. Two assessment tasks were performed using a Likert scale with a range of 1 to 4 with 0.5 increments as shown on Tables 1 and 2. If there was a major score difference of 1.5 or more, a consensus review was held to analyze the factors that contributed to the major differences and to reach a consensus score.

**Table 1:**
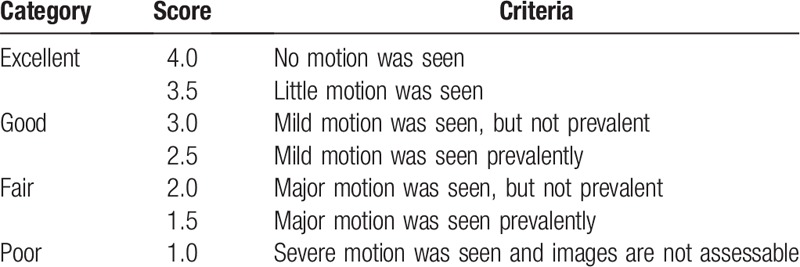
Likert scale for motion-based assessment of image quality.

**Table 2:**
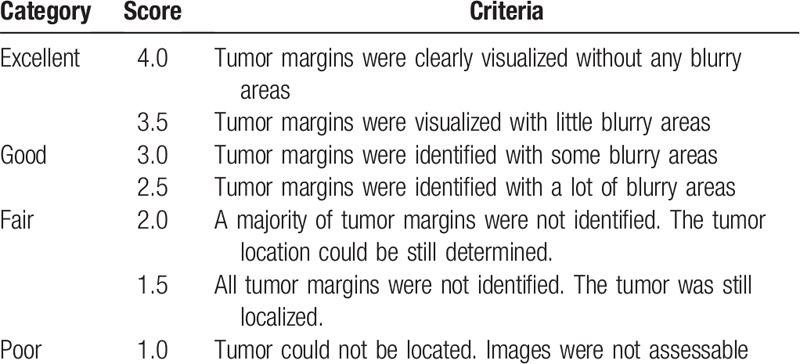
Likert scale for visualization-based assessment of image quality.

### 2.4 Statistical analysis

The scores were summarized using median and range, and compared between T2W data with and without MultiVane for each reviewer's assessment using the nonparametric signed-rank test. *P* < .05 was considered to be statistically significant. All statistical analyses were conducted in SAS version 9.4 (SAS Institute, Cary, NC).

The values of kappa (*κ*) scores were used to assess interobserver variability between the 2 reviewers. We also calculated the mean score difference case-by-case between the 2 reviewers, noted as *δ* value, as follow:
 
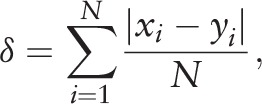


where *N* is 16 which is the total number of cases, *x_i_* and *y_i_* are respectively the scores by reviewers 1 and 2 for case *i*.

### 2.5 Data availability statement

The data that support the findings of this study are available on request from the corresponding author (MVK). The data are not publicly available due to their containing information that could compromise the privacy of research participants.

## 3 Results

### 3.1 Severity of motion artifacts in bladder imaging

**Figure 1:**
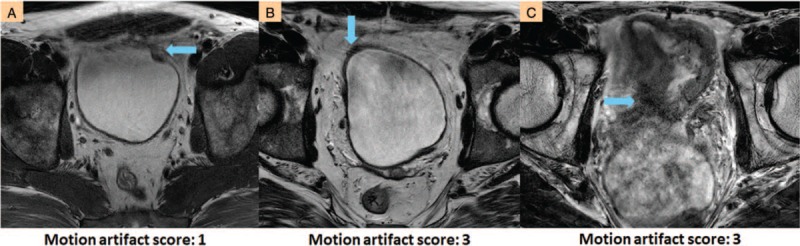
Motion artifacts are caused by respiration and peristalsis in bladder imaging. (A and B) Respiratory artifacts on the bladder. (C) Peristalsis-induced artifacts on the bladder. In A, the bladder tumor is obscured by a number of streaks from breathing. Only a few streaks overlap on the bladder lesion in B. Peristalsis interrupts the tumor margin in C. The arrows indicate where the artifacts overshadow on the bladder wall.

**Figure 2:**
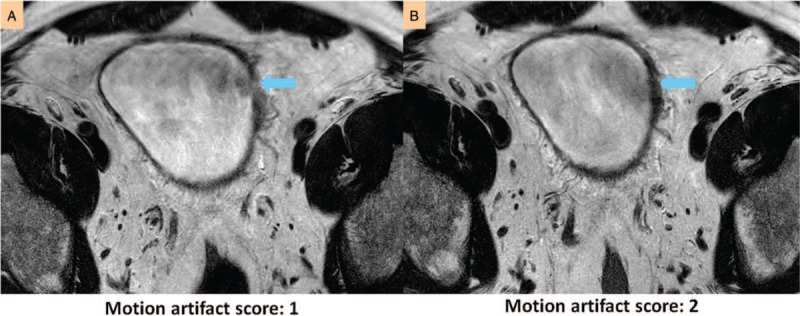
Severity of motion artifacts is different within a patient from scan 1 (A) to scan 2 (B) without MultiVane. (A) Motion artifacts could lead to misinterpretation of tumor location. (B) Motion artifacts are mild and the tumor can be located.

Artifacts induced by respiration were observed in 14 scans and by peristalsis in 2 scans. These artifacts degraded the image quality and interfered with the visualization of the bladder wall and tumor. The severity of the motion artifacts varied among patients (Fig. 1) and were predominantly due to involuntary, physiologic motions. The severity also differed between scans for the same patient (Fig. 2).

The median scores of motion and visualization assessments for MR images without MultiVane were 2.0 and 2.0 for reviewer 1, and 1.5 and 1.5 for reviewer 2 (Table 3). The motion-induced artifacts degraded the image quality, overlapped bladder wall and tumor, and thus sometimes caused the misinterpretation of tumor location.

**Table 3:**
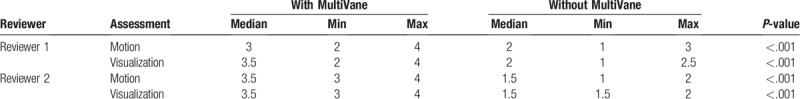
Scores for motion and visualization assessments with and without MultiVane.

### 3.2 Improving image quality with MultiVane (PROPELLER)

**Figure 3:**
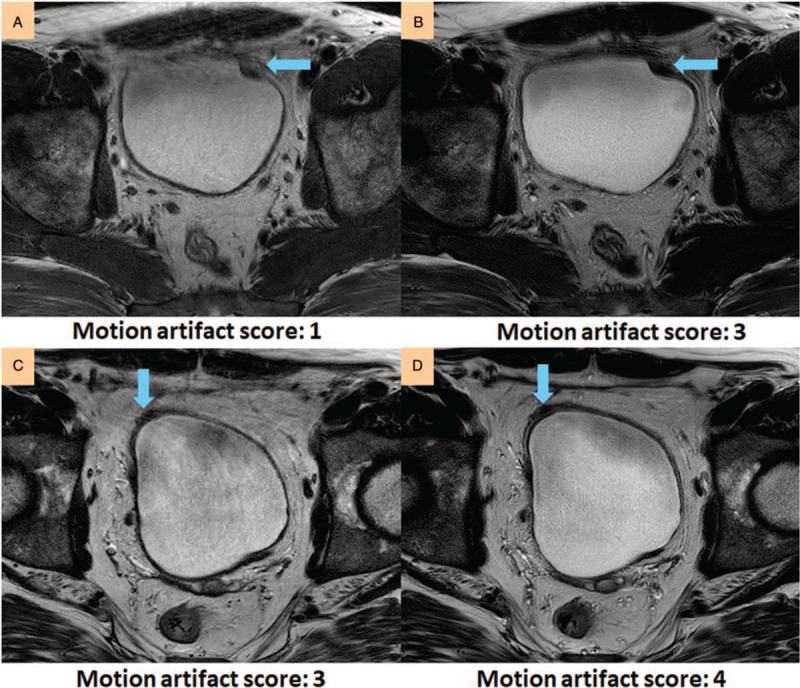
Improvement in respiratory artifacts. (A and B) Severe respiratory artifacts without MultiVane (A) are substantially reduced with MultiVane (B). (C and D) All breathing-induced streaks without MultiVane (C) are eliminated with MultiVane (D). The arrows indicate where the artifacts affect the bladder and how they are resolved with MultiVane.

**Figure 4:**
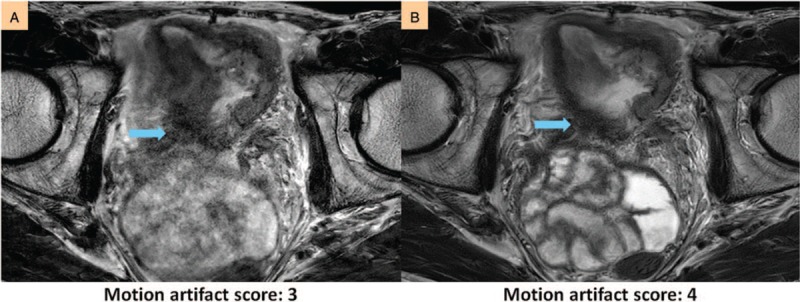
Improvement in peristalsis-induced artifacts. The obscurity of tumor margins without MultiVane (A) was significantly improved with MultiVane (B). The arrows indicate where the artifacts affect the bladder and how they are resolved with MultiVane.

**Figure 5:**
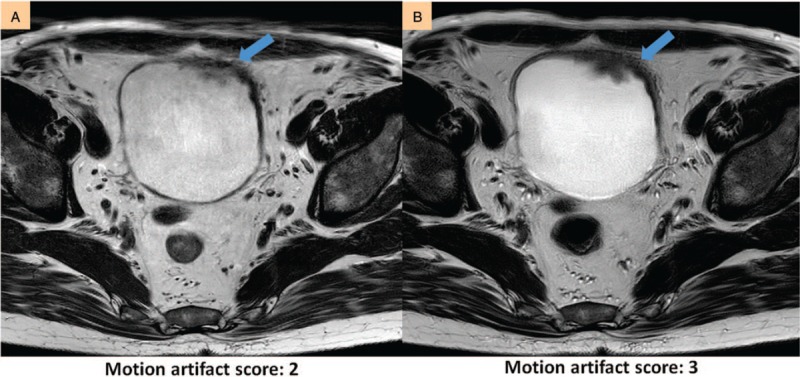
Improvement in tumor visualization. (A) Tumor location can be seen, but the margins cannot be visualized (visualization score: 2) without MultiVane. (B) Tumor margins are delineated with MultiVane (visualization score: 4).

The application of PROPELLER substantially reduced the artifacts induced by both respiration (Fig. 3) and peristalsis (Fig. 4). The improvement was seen in all cases with different levels of motion severity (Fig. 3). The peristalsis-induced artifacts were resolved on T2W images with MultiVane (Fig. 4). The median motion and visualization scores increased to 3 and 3.5 for reviewer 1, and 3.5 and 3.5 for reviewer 2. Bladder tumor margins were better delineated in all cases (Fig. 5). Nonparametric signed-rank test showed that both motion artifact and tumor visualization were significantly (both *P* < .001) improved. Both reviewers independently had this finding.

There was slight change in the signal contrast and morphologic shapes on T2W images with MultiVane. However, it had no impact on the diagnostic tasks performed in the study.

### 3.3 Reader preference bias and inter-observer variability

We found that the mean score difference for motion assessment without MultiVane (*δ* = 0.41) was the same as with MultiVane. That was also true for the visualization scoring where the *δ* values were 0.50 for both T2W data with and without MultiVane.

Out of 64 assessment scores from each reviewer, 24 (38%) were the same (absolute agreement), 26 (41%) revealed a difference of only 1 increment (0.5, strong agreement), 11 (17%) with a difference of 2 increments (1.0, intermediate agreement), and 3 (5%) with differences of 1.5 or 2 (little agreement). Scoring differences occurred predominately due to reader preference reflecting slightly more favorable scoring. This was found for example in a case when the first reviewer assigned a score of 3 (mild motion artifacts) while the second reviewer gave a score of 3.5 (little motion artifacts) for T2W images with MultiVane. The same case without MultiVane was scored 1 by the first reviewer and 1.5 by the second reader. Therefore, even though the preference bias resulted in *κ* scores of less than 0.2 for all assessments, the independent reviews still reached the agreement on the significant improvement of image quality (as detailed in the previous section).

A consensus review was performed to analyze the factors that contributed to the 3 score differences of 1.5 or 2.0. Differences occurred in cases in which there was only diffuse bladder wall thickening, instead of a bulky tumor. Some parts of the thickening were in the motion-affected areas, while other parts were clear of artifacts. One reviewer had focused on the whole bladder image quality while the other reader assigned scores based on the tumor image quality.

## 4 Discussion

In pelvic imaging, many patients, due to their health condition, are unable to maintain a shallow regular breath during an MRI sequence. As a result, MR images of the bladder are often degraded by unavoidable breathing motion as the bladder is close to the superficial abdominal fat. Respiratory motion artifact was previously reported in pelvic imaging.^[2]^ There has not been any satisfactory noninvasive solution to this issue. Our data have shown that the application of PROPELLER can resolve respiratory motion artifacts of different severity.

Peristalsis is an involuntary motion of the GI track. Depending on patient anatomy, peristalsis may directly affect the bladder region on MRI. In this study, there were 2 scans in which peristalsis-induced artifacts were seen to disrupt the visualization of the bladder tumors. We have demonstrated that PROPPELLER acquisition could remove the artifact completely or substantially reduce it improved morphologic visualization.

Several studies have previously reported on the application of PROPELLER in pelvic MR imaging.^[10,18]^ These studies, which mainly focused on the visualization of female organs and gynecological lesions, all concluded that PROPELLER could reduce artifacts and improve image quality. Nonetheless, the study by Fujimoto et al found no statistical significance between T2W with and without PROPELLER for the visualization of bladder organ. It is noteworthy that in this study bladder was imaged at 1.5T and not at the center of FOV. Meanwhile, our data with the focus on bladder imaging showed a significant improvement of bladder wall visualization using PROPELLER at 3T.

In theory, PROPELLER can correct any type of motion in MRI data by eliminating inconsistent sampled data due to patient motion. Two studies by Dietrich et al and Nagatomo et al concluded that PROPELLER could reduce the motion artifacts in anatomical shoulder MRI.^[5,13]^ Bayramoglu et al and Hirokawa et al reported that PROPELLER was an effective approach to improve breathing motion artifacts for better image quality in T2W-MRI of the upper abdomen.^[3,8]^ Other studies showed that the method also improved the anatomical imaging (T1W or/and T2W) of cardiac, lungs, and head and neck by motion reduction.^[6,9,12,14]^ Our study is the first to report that the severity of respiration and peristalsis induced artifacts on bladder imaging can be compensated by using PROPELLER, which appears to be a valuable tool to ensure consistent diagnostic image quality.

Improvement of lesion or disease visualization is one of the most important clinical values of PROPELLER that has been reported. Meier-Schroers et al indicated that MultiVane could provide high image quality to improve the detection of lung lesions.^[12]^ In MRI of the female pelvis, Lane et al presented that imaging with PROPELLER was superior for delineation of ovarian borders and follicles as well as detection of fibroids.^[10]^ Eriksson et al showed that the application of PROPELLER had excellent tissue contrast in brain imaging and could visualize the internal hippocampal structures and tissue changes associated with hippocampal sclerosis. In our study, T2W imaging with PROPELLER showed substantial improvement in delineating bladder tumors and their margins.

Dietrich et al indicated that PROPELLER increased the scan time. Lane et al reported that PROPELLER only has a slightly longer scan time in female pelvic imaging. Hirokawa et al demonstrated that it is possible to reduce image artifacts and obtain better image quality with the same scan time and coverage with PROPELLER. Our results also indicated that the application of this k-space sampling technique can significantly reduce motion-induced artifacts and provides high-quality imaging of the morphology of the bladder as well as the pelvis while similar coverage, scan time and image contrast can still be achieved.

The utilization of a Likert scale in this study showed that reviewers had their own preferences for categorizing image quality that led to different baseline preference while scoring. However, the *δ* values and the case-by-case assessment showed that the reviewers applied their preferences consistently throughout the assessment of the data with or without MultiVane. Therefore, regardless of the inter-observer variability shown by kappa scores, both reviewers found a significant difference in the image quality improvement of *P* < .001 for both motion artifacts and tumor visualization. They both recommended the use of the PROPELLER technique for standard of care bladder MRI. Hence, it should be noted that while kappa values are often used to evaluate the inter-observer agreement, they may overestimate the variability with a Likert scale. As long as reviewers are consistent in their assessment, the preference bias will not prevent them from reaching the same conclusion as was seen with the significant improvement achieved with MultiVane in this study. The 3 large score differences (1.5 or 2) suggested that comprehensive instructions and training datasets should be used to minimize reader bias in relation to variation of anatomy and disease characteristics.

The study had several limitations. We noted a slight change in the signal contrast and morphologic shapes, and little random noise with the PROPELLER acquisition. However, the reviewers agreed that these changes did not affect the diagnosis and would be outweighed by the benefits of motion artifact reduction. Due to this slight, but distinctly visible change and the obvious reduction in motion artifacts, our reviewers pointed out that they always could identify which one was acquired with or without MultiVane (PROPELLER). We, therefore, concluded that randomization or not would not make a difference in the final results because the readers would have never been truly blinded. Thus, we decided to not anonymize the data and presented the data as we did. Our assessment included only 16 intra-individual bladder MRI comparisons in patients with identified motion on the conventional imaging. However, we consistently observed a substantial improvement in motion artifacts and tumor visualization in all cases with PROPELLER acquisition. With this observation, further studies will be conducted to assess the diagnostic improvement of PROPELLER in tumor staging and monitoring therapeutic response of bladder cancer using pathological findings as a reference standard.

## 5 Conclusions

The PROPELLER data acquisition significantly reduced motion artifacts and thereby improved the visualization of bladder tumors. We propose that clinical T2W MRI of the bladder should utilize this motion compensation approach to ensure consistent image quality for data assessment.

## Author contributions

**Conceptualization:** Huyen Thanh Nguyen, Michael Vinzenz Knopp.

**Formal analysis:** Huyen Thanh Nguyen, Zarine Ketul Shah, Michael Vinzenz Knopp.

**Funding acquisition:** Michael Vinzenz Knopp.

**Investigation:** Huyen Thanh Nguyen, Zarine Ketul Shah, Amir Mortazavi, Kamal S. Pohar, Michael Vinzenz Knopp.

**Methodology:** Huyen Thanh Nguyen, Zarine Ketul Shah, Amir Mortazavi, Kamal S. Pohar, Lai Wei, Debra Lyn Zynger, Michael Vinzenz Knopp.

**Project administration:** Huyen Thanh Nguyen.

**Resources:** Huyen Thanh Nguyen, Zarine Ketul Shah, Amir Mortazavi, Kamal S. Pohar, Lai Wei, Debra Lyn Zynger, Michael Vinzenz Knopp.

**Software:** Huyen Thanh Nguyen.

**Supervision:** Huyen Thanh Nguyen, Zarine Ketul Shah, Amir Mortazavi, Kamal S. Pohar, Michael Vinzenz Knopp.

**Validation:** Huyen Thanh Nguyen, Zarine Ketul Shah, Michael Vinzenz Knopp

**Visualization:** Huyen Thanh Nguyen, Zarine Ketul Shah.

**Writing – original draft:** Huyen Thanh Nguyen.

**Writing – review and editing:** Huyen Thanh Nguyen, Zarine Ketul Shah, Amir Mortazavi, Kamal S. Pohar, Lai Wei, Debra Lyn Zynger, Michael Vinzenz Knopp.

Huyen Thanh Nguyen orcid: 0000-0002-4409-0824.

Huyen Thanh Nguyen orcid: 0000-0002-4409-0824.
